# Supplementary material for: Anti-Obesity Effect of Different Opuntia stricta var. dillenii’s Prickly Pear Tissues and Industrial By-Product Extracts in 3T3-L1 Mature Adipocytes
Source: Nutrients. 2024 Feb 9;16(4):499. doi: 10.3390/nu16040499 (PMC10892177; doi:10.3390/nu16040499)
Supplement: Supplementary file 1 [file nutrients-16-00499-s001.zip › Supplementary material/Table S2.pdf]

Table S2. Antioxidant activities (ORAC and LOX-FL) of *Opuntia stricta var. dillenii*'s whole fruit, tissues (peel, and pulp) and by-product (bagasse).

Biological activity	*Opuntia strcita* var. *Dillenii*			
	Whole fruit	Peel	Pulp	Bagasse
**Antioxidant (µmol Trolox eq/g Dry weight)**				
ORAC	219.56 ± 17.09[a]	122.97 ± 0.73[b]	255.04 ± 3.81[a]	211.31 ± 0.47[a]
LOX-FL	429.51 ± 54.64[a]	404.12 ± 42.27[a]	480.50 ± 22.83[a]	438.15 ± 8.67[a]

Results were expressed as mean ± standard deviation (n = 3). This came from obtaining at least two independent extracts (n = 2) and performing the biological activity determinations of each time (n = 2). Superscript letters indicate statistically significant differences (p ≤ 0.05) between different OPD tissues
