# Supplementary material for: Anti-Obesity Effect of Different Opuntia stricta var. dillenii’s Prickly Pear Tissues and Industrial By-Product Extracts in 3T3-L1 Mature Adipocytes
Source: Nutrients. 2024 Feb 9;16(4):499. doi: 10.3390/nu16040499 (PMC10892177; doi:10.3390/nu16040499)
Supplement: Supplementary file 1 [file nutrients-16-00499-s001.zip › Supplementary material/Table S3.pdf]

Table S3. Effects of 10, 25, 50 and 100 μg/mL of extracts from *Opuntia stricta* var. *dilenii* whole fruit, peel, pulp and bagasse tissues on triacylglycerol content (%) of 3T3-L1 mature adipocytes treated for 24 h

Tryglyceride content (%)				
	Opuntia stricta* var. *dilenii			
**Doses**	**Whole fruit**	**Peel**	**Pulp**	**Bagasse**
10 μg/mL	84.38 ± 10.75 ^{cb}	120.65 ± 4.14^{a}	89.55 ± 6.52^{a}	111.52 ± 4.13^{a}
25 μg/mL	107.08 ± 10.22^{a}	90.08 ± 14.52^{a}	85.36 ± 4.86^{a}	85.18 ± 5.34^{a}
50 μg/mL	72.63 ± 7.20^{c*}	76.39 ± 5.72^{b*}	62.65 ± 2.48^{b*}	73.37 ± 9.42^{a}
100 μg/mL	65.44 ± 8.46^{c*}	78.09 ± 4.80^{b*}	65.84 ± 2.43^{b*}	88.10 ± 10.31^{a}

Control represent 100% of accumulation. Results were expressed as mean ± standard deviation (n = 3). Superscript letters indicate statistically significant differences ($p \leq 0.05$) between treatments in the same tissue. (*) Indicates significant differences ($p \leq 0.05$) between different OPD tissues and control.
