# Supplementary figures and images for: Anti-Obesity Effect of Different Opuntia stricta var. dillenii’s Prickly Pear Tissues and Industrial By-Product Extracts in 3T3-L1 Mature Adipocytes
Source: Nutrients. 2024 Feb 9;16(4):499. doi: 10.3390/nu16040499 (PMC10892177; doi:10.3390/nu16040499)

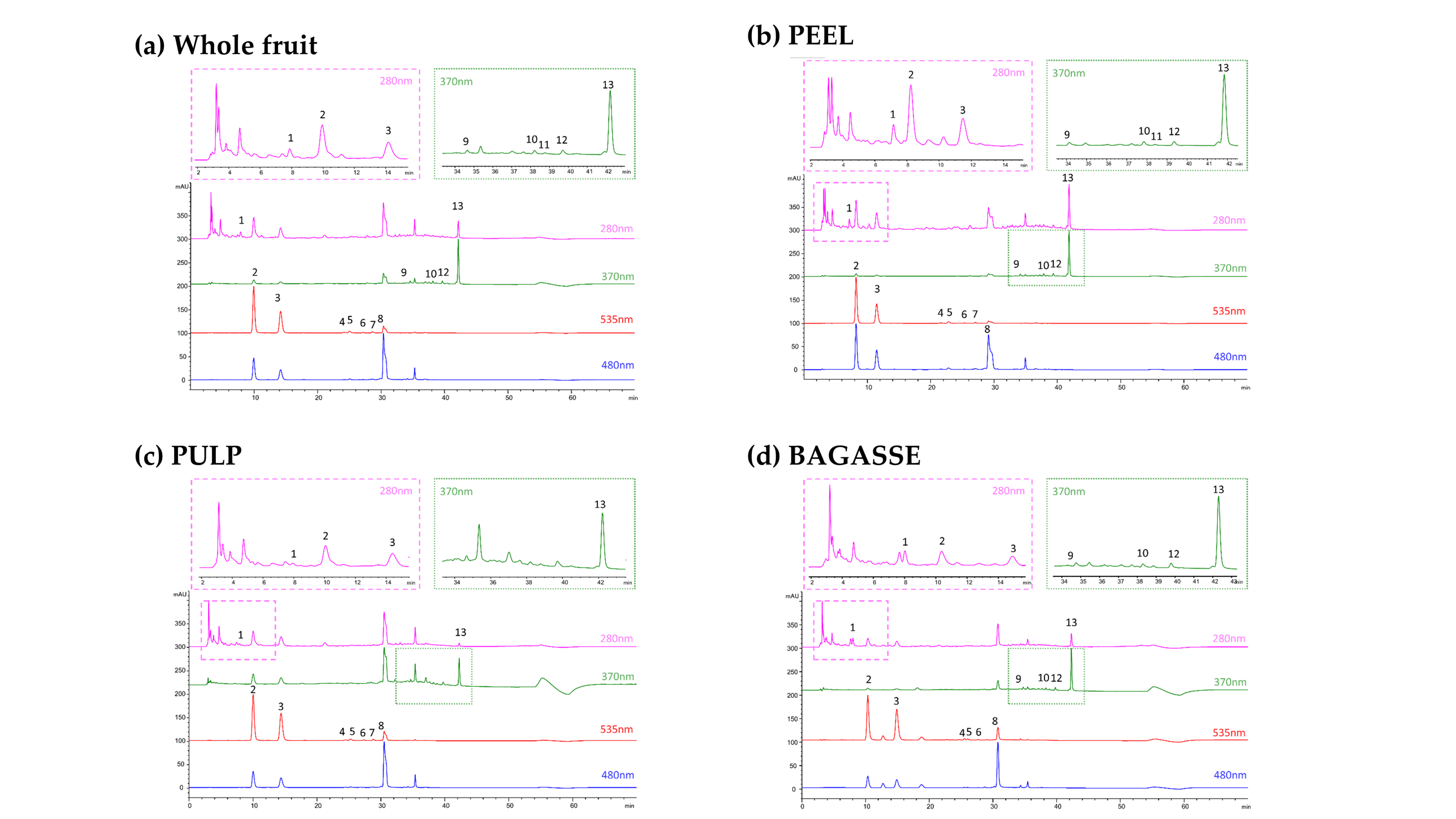

Supplement: Supplementary file 1 [file nutrients-16-00499-s001.zip › Supplementary material/Figure S1.tif]

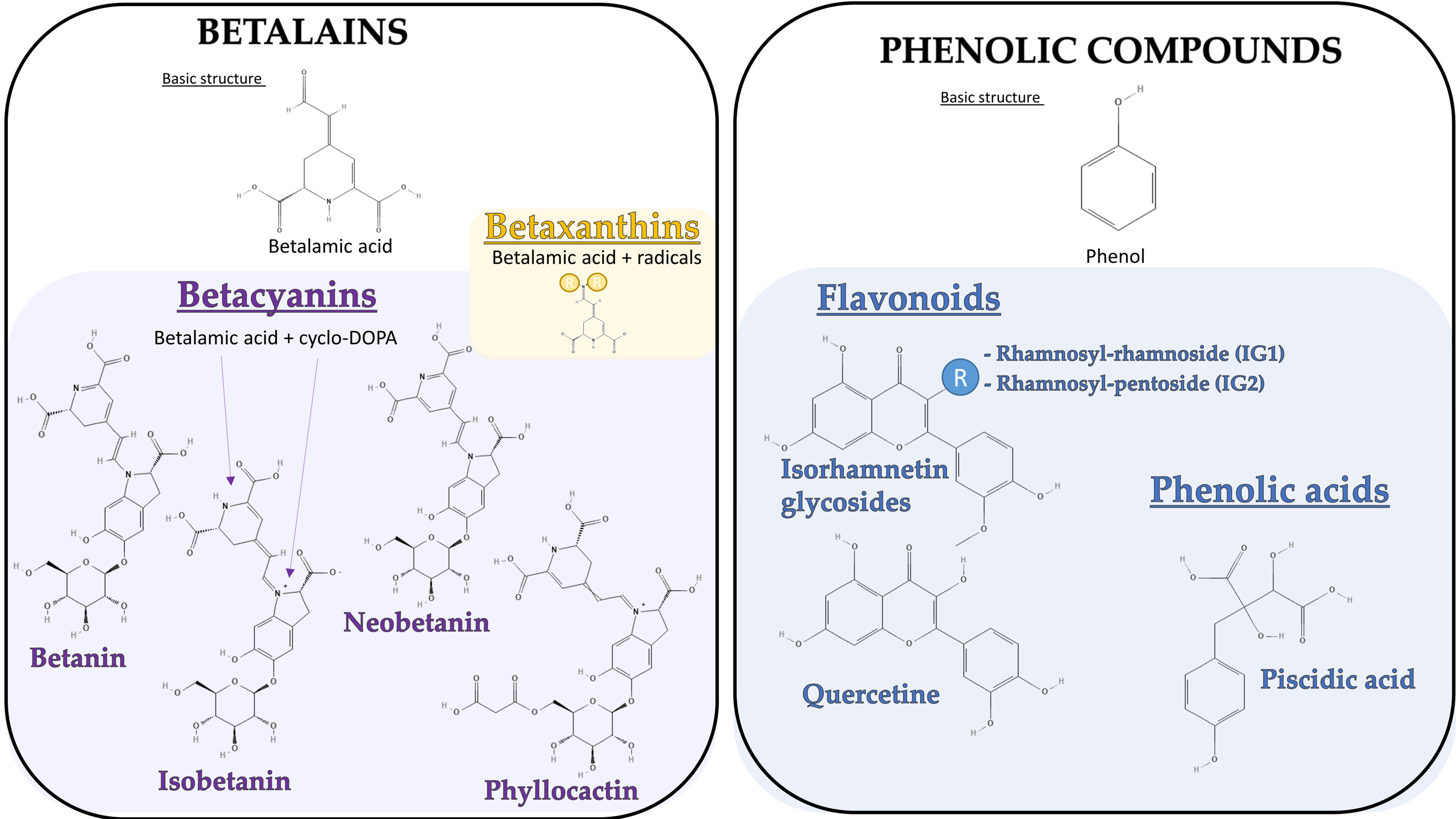

Supplement: Supplementary file 1 [file nutrients-16-00499-s001.zip › Supplementary material/Figure S2.tif]
